# Extracranial Abscopal Responses after Radiation Therapy for Intracranial Metastases: A Review of the Clinical Literature and Commentary on Mechanism

**DOI:** 10.7759/cureus.4207

**Published:** 2019-03-08

**Authors:** Chengcheng Gui, Lawrence R Kleinberg, Michael Lim, Kristin J Redmond

**Affiliations:** 1 Radiation Oncology and Molecular Radiation Sciences, The Johns Hopkins University School of Medicine, Baltimore, USA; 2 Neurosurgery, The Johns Hopkins University School of Medicine, Baltimore, USA

**Keywords:** brain mets, radiation oncology, stereotactic radiosurgery, whole brain radiation therapy, cancer immunology, abscopal effect

## Abstract

The current literature contains a small number of case series and individual case reports that describe radiographic regression of extracranial tumors after treatment of one or more brain metastases with radiation therapy. These observations suggest an abscopal effect that traverses the blood-brain barrier. The purpose of this review is to describe the clinical evidence for this phenomenon and potential mechanistic relationships between radiation, the blood-brain barrier, and the abscopal effect. Among reported cases, the majority of patients received systemic immunotherapy, which is consistent with an immunologic mechanism underlying abscopal responses. Preclinical data suggest that radiation may play multiple roles in this process, including the release of tumor-associated antigens and disruption of the blood-brain barrier. Future studies investigating the abscopal effect would benefit from more rigorous methods to control for patient and treatment factors that may affect distant tumor response.

## Introduction and background

The abscopal effect of radiation therapy can be defined as tumor regression caused by irradiation of a distant site. Although observations consistent with the abscopal effect are rare, there are several reasons for the growing interest in this phenomenon. Prominent among these is the increasing use of immunotherapy in conjunction with radiation therapy. The mechanisms of the abscopal effect, while not completely understood, are thought to be mediated by immunogenic elements [1-3]. Thus, an improved understanding of the abscopal effect and its immunologic correlates has implications for the synergistic use of radiation therapy and immunotherapy.

In particular, there is increasing evidence for combined radiation therapy and immunotherapy in the treatment of brain metastases, including in patients with metastatic melanoma [4-5]. A small number of studies have described extracranial tumor response following radiation therapy for brain metastases, predominantly in patients who also received systemic immunotherapy [4,6-17]. However, despite the special relationship between the central nervous system and the immune system resulting from the blood-brain barrier, there has not been a substantial discussion about the mechanisms that would allow the immunologic correlates of the abscopal effect to traverse this boundary.

Thus, the purposes of this review were to (1) assess the current clinical evidence for extracranial abscopal responses originating from radiotherapeutic treatment of brain metastases; (2) summarize, based on the preclinical literature, the relationships between the immunologic mechanisms of the abscopal effect, the blood-brain barrier, and brain irradiation; and (3) reassess the significance of the clinical evidence with a basic mechanistic framework in mind.

## Review

Clinical cases of extracranial abscopal responses after irradiation of brain metastases

A PubMed search was performed with an end date of December 2018, using the terms “abscopal”, “brain”, and “radiation”. This search yielded 25 results, which were assessed for further review. In addition, a Google Scholar search was performed, using the same terms and end date but limited to studies published during or after 2013. This second search yielded 2170 results, of which the 200 most relevant results were assessed for further review. Clinical case series and individual case reports were included for further review if the authors described an extracranial abscopal response in at least one patient who received radiation therapy for one or more brain metastases. Clinical studies were excluded if no patients had brain metastases treated with radiation therapy and subsequent evidence of an extracranial abscopal response. Preclinical studies and literature reviews identified by this search were also excluded from comparative review.

Thirteen studies published between 2013 and 2018 describing 53 cases consistent with an extracranial abscopal response following radiation therapy for brain metastases were reviewed (Table 1). The majority of studies evaluated possible abscopal responses in terms of radiographic regression of extracranial tumor burden. Two of the larger case series further calculated a “delta-delta”, defined as the difference in rate of radiographic change in tumor size before and during radiation therapy [6,12]. Tumors that regressed at faster rates during radiation therapy compared to prior were considered as more likely to be experiencing an abscopal response. One case report recognized a possible abscopal response by an increase in tumor-specific antibodies [13]

**Table 1:** Thirteen clinical studies with evidence suggesting an extracranial abscopal response after radiation therapy for one or more brain metastases “Delta-delta”: change in the rate of radiographic response before and during radiation therapy; an increase in the rate of tumor regression is suggestive of an abscopal response. SRS: stereotactic radiosurgery. WBRT: whole brain radiation therapy.

First author	Year	Reference	Primary histology	Number of relevant cases	Evidence of abscopal response	Location of abscopal response	Modality of radiation therapy	Systemic therapy
Chandra	2015	6	Melanoma	23	“Delta-delta”	Various	SRS, WBRT	Ipilimumab
Chuang	2018	7	Colorectal adenocarcioma	1	Radiographic response	Lung	WBRT	None
Galkin	2018	8	Melanoma	1	Symptomatic	Skin	SRS	None
Grimaldi	2014	9	Melanoma	7	Radiographic response	Various	SRS, WBRT	Ipilimumab
Katayama	2017	10	Non-small cell lung cancer	1	Radiographic response	Lung	WBRT	None
Kiess	2015	4	Melanoma	1	Radiographic response	Pelvis, lung	SRS	Ipilimumab
Ruzevick	2013	11	Melanoma	1	Radiographic response	Liver, extremity	SRS	Ipilimumab
Schoenfeld	2015	12	Melanoma	11	“Delta-delta”	Various	SRS, WBRT	Ipilimumab
Stamell	2013	13	Melanoma	1	Anti-MAGEA3 antibodies, response to cancer antigen PASD1	Skin	SRS	Ipilimumab
Sullivan	2013	14	Melanoma	1	Radiographic response	Pelvis, spine	SRS	Vemurafenib
Thallinger	2015	15	Melanoma	1	Radiographic response	Renal, lung, liver	WBRT	Ipilimumab
Theurich	2016	16	Melanoma	3	Radiographic response	Various	SRS, WBRT	Ipilimumab
Yarchoan	2015	17	Non-small cell lung cancer	1	Radiographic response	Lung, liver, adrenal	SRS	Cisplatin, pemetrexed

The cases presented in these studies were highly homogeneous in terms of the disease and treatment modalities. Forty-eight of 53 patients were diagnosed with metastatic melanoma and received a combination of radiation therapy and ipilimumab. Three patients with melanoma, non-small cell lung cancer, and likely colorectal adenocarcinoma metastatic to the brain experienced an abscopal response with radiation therapy alone, without any systemic therapy [7-8,10]. In addition, abscopal responses were observed in one patient with melanoma treated with vemurafenib and one patient with non-small cell lung cancer treated with cytotoxic chemotherapy [14,17].

However, there was no clear consensus among the larger case series on whether the timing of immunotherapy with respect to radiation therapy influences the abscopal effect. Schoenfeld et al. found that patients with brain metastases who received immunotherapy within three months before or after radiation therapy were more likely to experience an extracranial tumor response (63% versus 7%, p = 0.003) [12]. By contrast, Chandra et al. and Grimaldi et al. did not find significant relationships between distant tumor response and timing of immunotherapy with respect to radiation therapy [6,9]. However, their cohorts were more heterogeneous than that of Schoenfeld et al., as they also included patients who received radiation therapy for extracranial metastases. In addition, the authors do not clearly explain how they defined the timing of immunotherapy as a variable for analysis, which could also potentially explain their differences in results compared to Schoenfeld et al.

Similarly, it is not clear whether fractionation affects the likelihood of an abscopal response. Chandra et al. described greater rates of distant tumor response among patients treated with fractions less than or equal to 3 Gy compared to greater than 3 Gy, when assessing their combined cohort of patients who received radiation therapy for intracranial or extracranial metastases [6]. Despite this isolated finding, both stereotactic radiosurgery and whole brain irradiation have preceded abscopal responses, suggesting that the mechanism is not necessarily limited by fractionation schedule [6-17].

Immunologic mechanisms of the abscopal effect

A large body of literature spanning two decades suggests that the abscopal response to radiation therapy is immunologic. In 1999, Chakravarty et al. demonstrated using a mouse model of Lewis lung carcinoma that radiation therapy and Flt-3 ligand, which stimulates the proliferation of dendritic cells, are synergistic in achieving systemic tumor control [18]. By contrast, Flt-3 ligand alone was ineffective, and the combination of radiation therapy and Flt-3 ligand was ineffective in T cell-deficient mice. In 2004, Demaria et al. presented similar findings in a mouse model of mammary carcinoma [1]. The work of Lugade et al. and Lee et al. provided further evidence that the relationship between radiation and increased antitumor immune response is mediated by greater levels of antigen presentation, followed by activation of T cells in tumor-draining lymph nodes and infiltration of the tumor by lymphocytes that recognize tumor-specific antigens [2-3].

In recent years, the synergy between radiation and immunotherapy has been observed clinically, as evidenced by the importance of delivering the two therapies concurrently or within a limited window of time [5,19]. Simultaneously, the preclinical theory of the immunologic effects of radiation has expanded rapidly. Bernstein et al. published a useful framework that categorizes the interactions between radiation and the immune system [20]. These categories include the release of tumor-associated antigens, induction of “danger” signals that allow the tumor antigens to be appropriately recognized, modulation of the tumor phenotype, and physical restructuring of the stroma and aberrant tumor vasculature, which allows immune cells to more easily access to the tumor [21-24].

**Figure 1:**
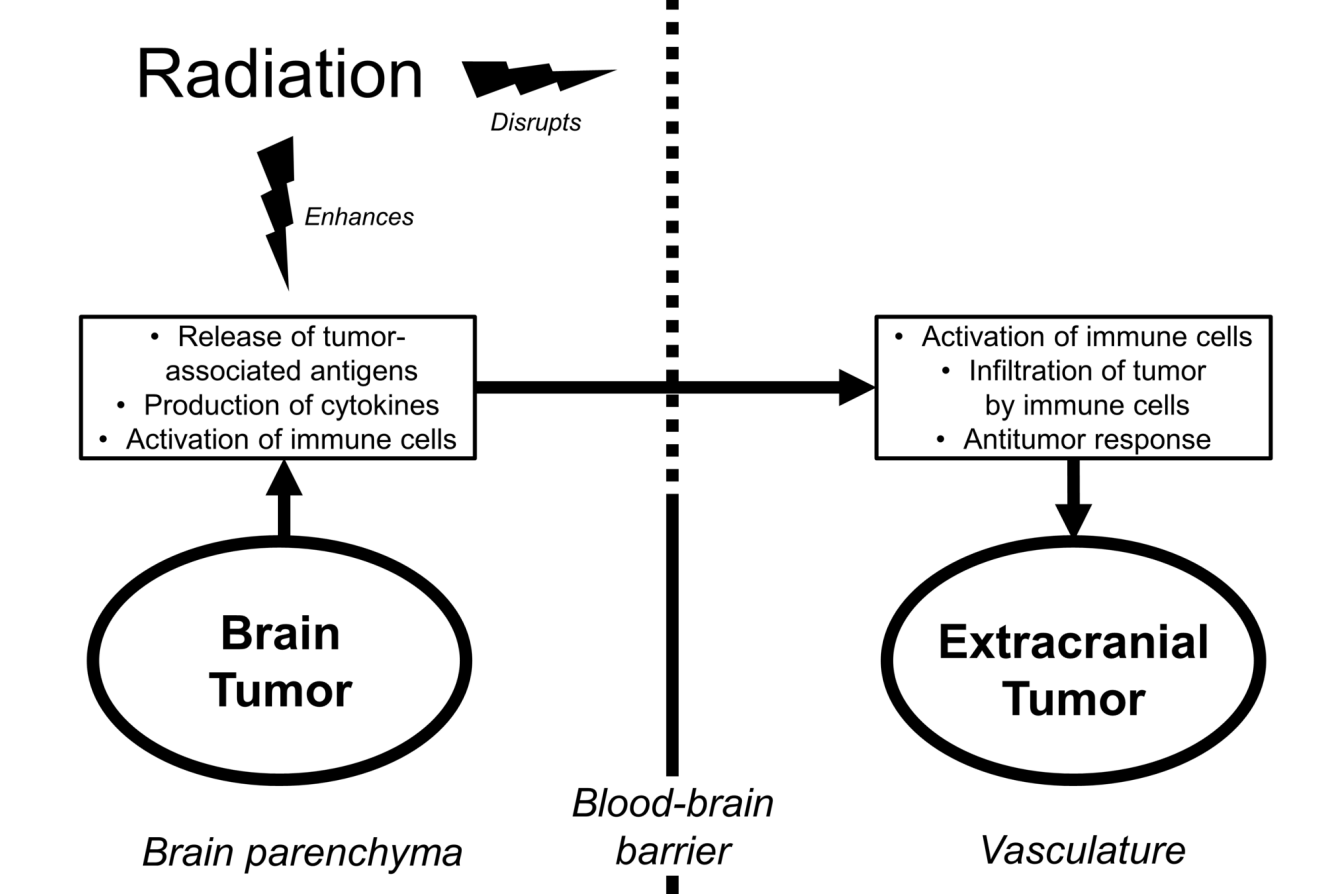
Schematic depicting an intracranial-to-extracranial abscopal response resulting from potential interactions between radiation, the blood-brain barrier, and antitumor immunity

Although some beneficial interactions between radiation therapy and the antitumor immune response are localized to the irradiated tumor itself, such as rearrangement of antigens from the interior of the tumor to the surface [20], there are numerous elements capable of acting at a distance, such as tumor-associated antigens, cytokines, and immune cells. These elements, individually or in combination, may be capable of mediating the abscopal effect (Figure 1).

Interaction between the blood-brain barrier and the abscopal effect

The central nervous system and the immune system share a unique relationship via the blood-brain barrier. In summary, this barrier consists of tight junctions that prevent all but the smallest lipophilic molecules (<400 Da and <8 hydrogen bonds) from diffusing freely, as well as a variety of differentially expressed carriers and receptor-mediated systems that closely control the transport of larger molecules [25-26]. The blood-brain barrier itself also secretes molecules that affect immunologic signaling, including cytokines, prostaglandins, and nitric oxide [27]. Immune cells may traverse the blood-brain barrier via diapedesis, but this occurs at low rates under physiological conditions [28]. Investigation of a mouse model suggests that a cell-mediated immune response to foreign antigens in the brain cannot be mounted unless the blood-brain barrier is disrupted [29].

At physiologic conditions, these components of the blood-brain barrier impede many of the possible immunologic mechanisms of the abscopal effect discussed previously. The low rate at which immune cells cross the blood-brain barrier is likely insufficient to produce tumor response to the extent observed in clinical cases. Oligopeptide antigens of suitable size to be presented to T cells are among the smaller elements that can mediate an abscopal response but nevertheless cannot diffuse freely across the blood-brain barrier due to the limitations on size and solubility [25]. As the transport of larger molecules is closely regulated, it is unlikely that tumor-associated antigens are able to cross in significant amounts via carrier- or receptor-mediated transport. This is analogous to the challenges of delivering therapeutic proteins across the blood-brain barrier [25].

Traversal of the blood-brain barrier by elements needed to produce an abscopal response may be substantially more feasible when the integrity of the blood-brain barrier is disrupted. Potential causes of disruption are diverse but may be broadly categorized as oxidative stress, as in ischemic stroke, imbalance among metabolites and signaling molecules, and inflammation [30-34]. In animal models and human cell lines, tumor necrosis factor α has been shown to increase permeability, possibly due to the reorganization of actin filaments [33-34]. Subsequently, failure of the blood-brain barrier may occur on both molecular and cellular levels, allowing permeability to larger and more hydrophilic molecules, such as tumor-associated antigens [30].

While it is plausible that the blood-brain barrier poses an obstacle to the abscopal effect and that its disruption increases the likelihood that the abscopal effect passes from the brain to the body, it is prudent to consider alternative pathways that run parallel to the vasculature. One such pathway is the “glymphatic” system, consisting of the interstitial fluid of the brain parenchyma, the cerebrospinal fluid, and the paravascular spaces around draining veins [35]. There is evidence that these components clear metabolic wastes, including glucose, lactate, and amyloid β [36]. Although rates of transport through this system are not well understood, its ability to clear amyloid suggests, at least, that tumor-associated oligopeptides are of a sufficiently small size to pass.

Effect of radiation on the blood-brain barrier

While the blood-brain barrier likely obstructs the abscopal effect from passing between intracranial and extracranial compartments, radiation therapy may play a role in disrupting this barrier. Multiple animal studies have described an increase in uptake of labeled markers following brain irradiation [37]. Potential mechanisms for this increased uptake include disruption of the vasculature leading to apoptotic cell death, the opening of tight junctions evidenced by actin stress fibers, and increased activation of vesicular transport pathways [38-41]. Furthermore, brain irradiation results in increased expression of a wide variety of acute phase reactants [42-43]. These include tumor necrosis factor α, which is understood to disrupt the blood-brain barrier [33-34].

Clinical evidence of radiation therapy disrupting the blood-brain barrier has been suggested by the detection of increased intracranial concentrations of radiolabeled markers and chemotherapy agents after irradiation. For example, Qin et al. observed a linear dose response when measuring uptake of a technetium marker during radiation therapy, as well as spatial differences in uptake between regions that received higher or lower doses [44]. The authors also described increased levels of methotrexate in the cerebrospinal fluid after at least 10 fractions of 2 Gy [45].

These observations present a possible explanation for why an abscopal effect traversing intracranial and extracranial compartments can be observed after radiation therapy. It is plausible that radiation not only increases the release of tumor-associated antigens but also facilitates their release into the extracranial vasculature (Figure 1).

Implications for interpretation of the clinical evidence

In summary, preclinical evidence suggests that the abscopal effect is mediated by immunologic elements that may include immune cells, cytokines, and tumor-associated antigens. Though not the sole pathway out of the brain, the blood-brain barrier likely presents a significant obstacle against the transport of these immunologic elements to the extracranial vasculature, and may consequently impede abscopal effects originating from intracranial tumors. However, there are known mechanisms by which radiation can disrupt the blood-brain barrier and thereby facilitate the transmission of the abscopal effect to the body (Figure 1). Understanding this, we are better equipped to evaluate the clinical evidence.

The fact that most patients who experienced an abscopal response after radiation for brain metastases also received systemic immunotherapy is consistent with the understanding that the abscopal effect is mediated by enhancement of the antitumor immune response. The prevalence of metastatic melanoma among the reported cases likely results from the early adoption of immunotherapy for this condition. For similar reasons, the observation of Schoenfeld et al. that the greater proximity of immunotherapy and radiation therapy in time correlates with greater rates of distant tumor response is also reasonable [12]. However, the uncertainty among other case series regarding the timing of modalities is not well-explained by preclinical theory, though it may be explained statistically by small and heterogeneous patient cohorts.

The observation that abscopal responses occur after both stereotactic radiosurgery and whole brain radiation therapy may also be explained mechanistically. Two possible mechanisms by which radiation induces the abscopal effect include the release of tumor-associated antigens and disruption of the blood-brain barrier. In cases of stereotactic radiosurgery, necrotic cell death in response to ablative doses results in the release of large amounts of tumor-associated antigens, which could pass through a disrupted blood-brain barrier to induce an abscopal effect at a distance. However, abscopal responses were also observed in cases of whole brain irradiation, which do not involve ablative doses. In these cases, it is plausible that disruption of the blood-brain barrier is the main mechanism by which radiation causes an abscopal effect, not rapid lysis of tumor cells. It may be that increasing the permeability of the blood-brain barrier could allow naturally occurring tumor antigens, potentially resulting from rapid tumor growth, to be released into the extracranial vasculature. Alternatively, a weakened blood-brain barrier could allow better access to the brain tumor by therapeutic agents or circulating immune cells, which could, in turn, lead to greater immune activation and distant tumor response. In these examples, an increased rate of tumor cell death directly due to radiation would not be necessary in explaining the abscopal effect.

Ishiyama et al. present an interesting case that supports the importance of the blood-brain barrier in understanding the abscopal effect [46]. The authors describe a patient with renal cell carcinoma who experienced spontaneous regression of lung metastases after stereotactic irradiation of bony lesions. Simultaneously, the patient’s brain metastases progressed, despite the regression of his somatic tumor burden. The implication is that the regression of the lung metastases represented an immunologically mediated abscopal effect that was prevented from reaching the brain. This is consistent with the case reports described in this review. While irradiation of the brain may allow the abscopal effect to reach the body, an undisrupted blood-brain barrier is expected to prevent the opposite process.

While most patients who experienced an abscopal response were treated with immunotherapy, there were a small number of individual cases in which abscopal responses were observed following radiation alone [7-8,10]. This is consistent with the prevailing theories of cancer immunosurveillance as an inherent function of the immune system [47]. Although checkpoint blockade is expected to increase the likelihood of observing an abscopal effect, it is not surprising to see a minority of cases that may reflect an already ongoing immune response.

Limitations of the current clinical evidence and future directions

In evaluating the abscopal effect, it is challenging to distinguish true distant responses to radiation from responses to systemic therapy. If the patient does not receive systemic immunotherapy agents, it may be possible to assess the immunologic changes that mediate to the abscopal effect, for example, by measuring titers of tumor-specific antibodies [13]. However, in cases of radiation therapy combined with systemic immunotherapy, accurate identification of the abscopal effect becomes even more difficult. If the abscopal effect of radiation is understood to be a result of enhancing the antitumor immune response, then one must effectively distinguish between the outcomes of two different immunologic therapies.

Two case series measured the abscopal effect using the difference in the rate of radiographic regression before and during radiation therapy, which they termed the “delta-delta” [6-12]. Although this methodology is appreciated as more quantitative than anecdotal reporting, it suffers from key weaknesses. First, the “delta-delta” method assumes approximately linear changes in tumor size over time, which may not reflect reality to an acceptable degree. Consequently, this method is limited by the length of time between imaging studies. Second, this method is potentially confounded by any other characteristic of the patient, treatment, or disease that may change over time and also affect the size of the tumor. For example, many of the case reports evaluated did not provide a detailed description of the timing of systemic immunotherapy in relation to radiation. Changes in systemic immunotherapy within the same time frame used to evaluate the “delta-delta” may severely confound the findings.

Ideally, abscopal responses will be evaluated in prospective studies with comparison groups. However, we recognize that such studies are difficult to conduct, particularly for rare outcomes. Assuming that it is necessary to assess the abscopal effect retrospectively, future studies would benefit from larger cohorts and greater quantitative rigor in controlling for potential confounding factors. As a hypothetical example, consider a retrospective study that characterizes in extensive detail the timing and extent of radiographic tumor response at all distant extracranial sites of metastasis, following irradiation of brain metastases in patients who either did or did not receive systemic immunotherapy. Such a study would ideally include a large consecutive cohort of patients treated at multiple institutions in order to control for confounding factors. To distinguish between the effects of systemic immunotherapy and radiation, it would be crucial to stratify by the timing of immunotherapy in relation to radiation therapy. In addition, the radiographic response should be measured in multiple ways, including the rate of volumetric regression over time, “delta-delta”, complete versus incomplete response, and duration of response.

## Conclusions

The current literature describes more than 50 clinical cases of extracranial tumor regression following radiation therapy for one or more intracranial metastases, suggesting an abscopal effect that crosses the blood-brain barrier. The majority of patients had metastatic melanoma and were treated with systemic immunotherapy in addition to radiation. Although it is not definite that these observations truly represent the abscopal effect of radiation, the temporal association between radiation therapy and tumor response is suggestive, and the preclinical literature plausibly suggests that radiation could play essential roles. These roles include the release of tumor-associated antigens from dying tumor cells and disruption of the blood-brain barrier to allow communication between the intracranial and extracranial compartments. Future studies assessing abscopal responses should seek to more rigorously control for factors that could affect distant tumor response, such as the timing of systemic immunotherapy.

## References

[1] Demaria S, Ng B, Devitt ML, Babb JS, Kawashima N, Liebes L, Formenti SC (2004). Ionizing radiation inhibition of distant untreated tumors (abscopal effect) is immune mediated. Int J Radiat Oncol Biol Phys.

[2] Lugade AA, Moran JP, Gerber SA, Rose RC, Frelinger JG, Lord EM (2005). Local radiation therapy of B16 melanoma tumors increases the generation of tumor antigen-specific effector cells that traffic to the tumor. J Immunol.

[3] Lee Y, Auh SL, Wang Y (2009). Therapeutic effects of ablative radiation on local tumor require CD8+ T cells: changing strategies for cancer treatment. Blood.

[4] Kiess AP, Wolchok JD, Barker CA (2015). Stereotactic radiosurgery for melanoma brain metastases in patients receiving ipilimumab: safety profile and efficacy of combined treatment. Int J Radiat Oncol Biol Phys.

[5] Lu VM, Goyal A, Rovin RA, Lee A, McDonald KL (2018). Concurrent versus non-concurrent immune checkpoint inhibition with stereotactic radiosurgery for metastatic brain disease: a systematic review and meta-analysis. J Neurooncol.

[6] Chandra RA, Wilhite TJ, Balboni TA (2015). A systematic evaluation of abscopal responses following radiotherapy in patients with metastatic melanoma treated with ipilimumab. Oncoimmunology.

[7] Chuang CH, Hsu JF, Shen YT, Yang CJ (2018). Regression of a metastatic lung mass after receiving whole brain irradiation: can the abscopal effect cross the blood-brain barrier?. Asia Pac J Clin Oncol.

[8] Galkin MV, Golanov AV, Vetlova E, Banov S, Kostjuchenko VV (2018). Advanced survival in patients with multiple irradiations for brain melanoma metastases and associated abscopal effect. Cureus.

[9] Grimaldi AM, Simeone E, Giannarelli D (2014). Abscopal effects of radiotherapy on advanced melanoma patients who progressed after ipilimumab immunotherapy. Oncoimmunology.

[10] Katayama K, Tamiya A, Koba T, Fukuda S, Atagi S (2017). An abscopal response to radiation therapy in a patient with metastatic non-small cell lung cancer: a case report. J Cancer Sci Ther.

[11] Ruzevick J, Nicholas S, Redmond KJ, Kleinberg LR, Lipson EJ, Lim M (2013). A patient with HIV treated with ipilimumab and stereotactic radiosurgery for melanoma metastases to the brain. Case Rep Oncol Med.

[12] Schoenfeld JD, Mahadevan A, Floyd SR (2015). Ipilmumab and cranial radiation in metastatic melanoma patients: a case series and review. J Immunother Cancer.

[13] Stamell EF, Wolchok JD, Gnjatic S, Lee NY, Brownell I (2013). The abscopal effect associated with a systemic anti-melanoma immune response. Int J Radiat Oncol Biol Phys.

[14] Sullivan RJ, Lawrence DP, Wargo JA, Oh KS, Gonzalez RG, Piris A (2013). Case records of the Massachusetts General Hospital. Case 21-2013. A 68-year-old man with metastatic melanoma. N Engl J Med.

[15] Thallinger C, Prager G, Ringl Ringl, H H, Zielinski C (2015). Abscopal-Effekt in der Therapie des malignen Melanoms (Abscopal effect in the treatment of malignant melanoma). Hautarzt.

[16] Theurich S, Rothschild SI, Hoffmann M (2016). Local tumor treatment in combination with systemic ipilimumab immunotherapy prolongs overall survival in patients with advanced malignant melanoma. Cancer Immunol Res.

[17] Yarchoan M, Lim M, Brahmer JR, Ettinger D (2015). Oligometastatic adenocarcinoma of the lung: a therapeutic opportunity for long-term survival. Cureus.

[18] Chakravarty PK, Alfieri A, Thomas EK (1999). Flt3-ligand administration after radiation therapy prolongs survival in a murine model of metastatic lung cancer. Cancer Res.

[19] Chen L, Douglass J, Kleinberg LR (2018). Concurrent immune checkpoint inhibitors and stereotactic radiosurgery for brain metastases in non-small cell lung cancer, melanoma, and renal cell carcinoma. Int J Radiat Oncol Biol Phys.

[20] Bernstein MB, Krishnan S, Hodge JW, Chang JY (2016). Immunotherapy and stereotactic ablative radiotherapy (ISABR): a curative approach?. Nat Rev Clin Oncol.

[21] Chen Z, Moyana T, Saxena A, Warrington R, Jia Z, Xiang J (2001). Efficient antitumor immunity derived from maturation of dendritic cells that had phagocytosed apoptotic/necrotic tumor cells. Int J Cancer.

[22] McBride WH, Chiang CS, Olson JL (2004). A sense of danger from radiation. Radiat Res.

[23] Kwilas AR, Donahue RN, Bernstein MB, Hodge JW (2012). In the field: exploiting the untapped potential of immunogenic modulation by radiation in combination with immunotherapy for the treatment of cancer. Front Oncol.

[24] Yu P, Rowley DA, Fu YX, Schreiber H (2006). The role of stroma in immune recognition and destruction of well-established solid tumors. Curr Opin Immunol.

[25] Pardridge WM (2012). Drug transport across the blood-brain barrier. J Cereb Blood Flow Metab.

[26] Sweeney MD, Zhao Z, Montagne A, Nelson AR, Zlokovic BV (2018). Blood-brain barrier: from physiology to disease and back. Physiol Rev.

[27] Muldoon LL, Alvarez JI, Begley DJ (2013). Immunologic privilege in the central nervous system and the blood-brain barrier. J Cereb Blood Flow Metab.

[28] Banks WA, Erickson MA (2010). The blood-brain barrier and immune function and dysfunction. Neurobiol Dis.

[29] Pollack IF, Lund RD (1990). The blood-brain barrier protects foreign antigens in the brain from immune attack. Exp Neurol.

[30] Obermeier B, Daneman R, Ransohoff RM (2013). Development, maintenance and disruption of the blood-brain barrier. Nat Med.

[31] Olmez I, Ozyurt H (2012). Reactive oxygen species and ischemic cerebrovascular disease. Neurochem Int.

[32] Fieschi C, Lenzi GL, Zanette E, Orzi F, Passero S (1980). Effects on EEG of the osmotic opening of the blood-brain barrier in rats. Life Sci.

[33] Deli MA, Descamps L, Dehouck MP, Cecchelli R, Joó F, Abrahám CS, Torpier G (1995). Exposure of tumor necrosis factor‐α to luminal membrane of bovine brain capillary endothelial cells cocultured with astrocytes induces a delayed increase of permeability and cytoplasmic stress fiber formation of actin. J Neurosci Res.

[34] Fiala M, Looney DJ, Stins M (1997). TNF-alpha opens a paracellular route for HIV-1 invasion across the blood-brain barrier. Mol Med.

[35] Bacyinski A, Xu M, Wang W, Hu J (2017). The paravascular pathway for brain waste clearance: current understanding, significance and controversy. Front Neuroanat.

[36] Ball KK, Cruz NF, Mrak RE, Dienel GA (2010). Trafficking of glucose, lactate, and amyloid-β from the inferior colliculus through perivascular routes. J Cereb Blood Flow Metab.

[37] Appelboom G, Detappe A, LoPresti M (2016). Stereotactic modulation of blood-brain barrier permeability to enhance drug delivery. Neuro Oncol.

[38] Bellinzona M, Gobbel GT, Shinohara C, Fike JR (1996). Apoptosis is induced in the subependyma of young adult rats by ionizing irradiation. Neurosci Lett.

[39] Shinohara C, Gobbel GT, Lamborn KR, Tada E, Fike JR (1997). Apoptosis in the subependyma of young adult rats after single and fractionated doses of X-rays. Cancer Res.

[40] Fauquette W, Amourette C, Dehouck MP, Diserbo M (2012). Radiation-induced blood-brain barrier damages: an in vitro study. Brain Res.

[41] Trnovec R, Kállay Z, Bezek S (1990). Effects of ionizing radiation on the blood brain barrier permeability to pharmacologically active substances. Int J Radiat Oncol Biol Phys.

[42] Hong JH, Chiang CS, Campbell IL, Sun JR, Withers HR, McBride WH (1995). Induction of acute phase gene expression by brain irradiation. Int J Radiat Oncol Biol Phys.

[43] Chiang C, McBride WH (1991). Radiation enhances tumor necrosis factor α production by murine brain cells. Brain Res.

[44] Qin DX, Zheng R, Tang J, Li JX, Hu YH (1990). Influence of radiation on the blood-brain barrier and optimum time of chemotherapy. Int J Radiat Oncol Biol Phys.

[45] Qin D, Ma J, Xiao J, Tang Z (1997). Effect of brain irradiation on blood-CSF barrier permeability of chemotherapeutic agents. Am J Clin Oncol.

[46] Ishiyama H, Teh BS, Ren H (2012). Spontaneous regression of thoracic metastases while progression of brain metastases after stereotactic radiosurgery and stereotactic body radiotherapy for metastatic renal cell carcinoma: abscopal effect prevented by the blood-brain barrier?. Clin Genitourin Cancer.

[47] Dunn CP, Old LJ, Schreiber RD (2004). The three Es of cancer immunoediting. Annu Rev Immunol.

